# Crescentic Glomerulonephritis Possibly Caused by COVID-19 Infection

**DOI:** 10.3390/jcm14103302

**Published:** 2025-05-09

**Authors:** Praveen Errabelli, Maulik Lathiya, Neeharik Mareedu, Loren P. Herrera Hernandez

**Affiliations:** 1 Allina Health, Minneapolis, MN 55407, USA; 2 Mayo Clinic, Rochester, MN 55905, USA; 3 UPMC, Cumberland, MD 21502, USA; neeharik.mareedu@gmail.com

**Keywords:** COVID-19, tubuloreticular inclusion body, crescentic glomerulonephritis

## Abstract

**Background:** The COVID-19 (coronavirus disease 2019) pandemic has presented a serious challenge to nephrologists, since it can lead to severe kidney injury in the form of acute tubular necrosis, with many patients requiring renal replacement therapy. This is predominantly seen in people who develop severe respiratory manifestations like ARDS (acute respiratory distress syndrome) from the viral infection, a cytokine storm or septic shock with unstable hemodynamics. It also presents with various glomerular injuries, mainly collapsing glomerulopathy in high-risk APOL1 (Apolipoprotein L1) genotype patients, thrombotic microangiopathy-related renal failure due to hyper coagulopathy and occasionally pauci-immune glomerulonephritis due to immune dysregulation. All the glomerular disorders that are caused by COVID-19 infection have been described under the designation COVAN (COVID-19-associated nephropathy). Proteinuria is a predominant presentation in glomerular disorders. Gross hematuria and AKI without any respiratory symptoms from COVID-19 infection have not been described so far in the literature. We are presenting one such rare case here. **Methods**: We have encountered a rare case of gross hematuria and severe acute renal failure. His serological work up was negative for all autoimmune etiologies that can cause Glomerulonephritis. He was found to have infection-related crescentic glomerulonephritis due to active COVID-19 infections discovered via kidney biopsy. He tested positive for SARS-CoV-2 but didn’t have any clinical respiratory symptoms. He has responded well to treatment with a steroid taper and antiviral medication (Remdesivir). This is a very rare renal manifestation of COVID-19 infection. **Results**: COVID-19 infection can result in crescentic glomerulonephritis. This can be diagnosed by kidney biopsy which shows extensive c3 deposits, tubuloreticular inclusion bodies along with crescentic lesions. This responds to treatment with steroids and Antiviral agents. Conclusions: Crescentic Glomerulonephritis should be considered as a possible etiology for severe acute kidney injury with hematuria in patients with active covid-19 infection without any respiratory symptoms. Kidney biopsy helps in diagnosing it and responds to treatment with steroids and antivirals.

## 1. Introduction

The involvement of kidneys in various systemic conditions is well-known. Infections often affect kidneys, and these infections most commonly cause sepsis, which leads to acute tubular necrosis/acute kidney injury from unstable hemodynamics. Infection-related glomerulonephritis is a well-known entity which is usually seen after severe acute bacterial infections mainly involving Staphylococcus and Streptococcus [1]. Viral infections leading to infection-related glomerulonephritis are relatively less common than bacterial infections. The recent COVID-19 pandemic has affected patients’ kidneys severely, with the most common kidney-related manifestation from this virus being acute tubular necrosis due to sepsis [2]. Another commonly described entity is collapsing glomerulopathy in high-risk APOL1 genotype patients [2]. Infection-related glomerulonephritis due to COVID-19 infection has not been described in detail so far. Here, we have described one such rare case and reviewed the literature of the various COVID-19-related kidney manifestations.

## 2. Case Presentation

A 69-year-old gentleman with obesity, hyperlipidemia, tobacco use, hypertension, psoriasis and irritable bowel syndrome presented to the emergency room with gross hematuria of a 1-week duration. He did not have any infections prior to this episode. He was admitted to the hospital because of AKI (acute kidney injury) and concerns about urinary tract infection. His blood pressure was 200/100 mm hg in the emergency room. He had normal renal function at baseline, with a creatinine level around 1 mg/dL and an EGFR (estimated glomerular filtration rate) around 75 mL/min. His urinalysis showed a large amount of blood, with >100 Red Blood Cells per high-power field, 20–30 White Blood Cells per high-power field and more than 300 mg/dL protein (Table S3). His Basic Metabolic Panel showed acute kidney injury with creatinine at 2.2 mg/dL and mild hypokalemia with serum potassium at 3.3 millimoles per L. His kidney ultrasound, renal artery Doppler study and CT (computed tomography) scan of his abdomen and pelvis without contrast were unremarkable. He received IV Rocephin initially in ED and was later switched to oral cephalexin due to a concern of pyelonephritis/urinary tract infection. His urine cultures a few days later showed a negative result. His hemoglobin was at 16 g/dL, his platelet count was 171 and his WBC count was 6.9 (Table S1). He was started on intravenous fluids, with a 50 mg home dose of atenolol daily; this was followed by 25 mg hydrochlorothiazide and then he was given 5 mg of amlodipine daily. His creatinine improved to 1.8 mg/dL after 24 h. His hematuria also improved. A urologist recommended outpatient evaluation with cystoscopy and a CT urogram for hematuria.

The patient was discharged and seen in the clinic a week later. During this visit, he reported severe fatigue, poor appetite, bilateral flank discomfort and persistent dark urine. The lab results showed severe acute renal failure with creatinine worsening up to 5.7 mg/dL (Figure S2), and a urinalysis showed a large amount of blood, >100 RBC per high-power, orange turbidity and 20–30 WBC per high-power field (Table S3). We could not test the urine for NGAL (neutrophil gelatinase-associated lipocalin) and Beta-2 Microglobulin as these are not offered at our laboratory. He was oliguric with a urine output of less than 500 cc per day and hypertensive with a blood pressure around 160/90 mm Hg. He was admitted to the hospital. His renal function continued to worsen, his creatinine worsened to 6.4 mg/dL, his BUN (blood urine nitrogen) was up to 96 mg/dL and he had persistent oliguria and hematuria.

**Figure 1:**
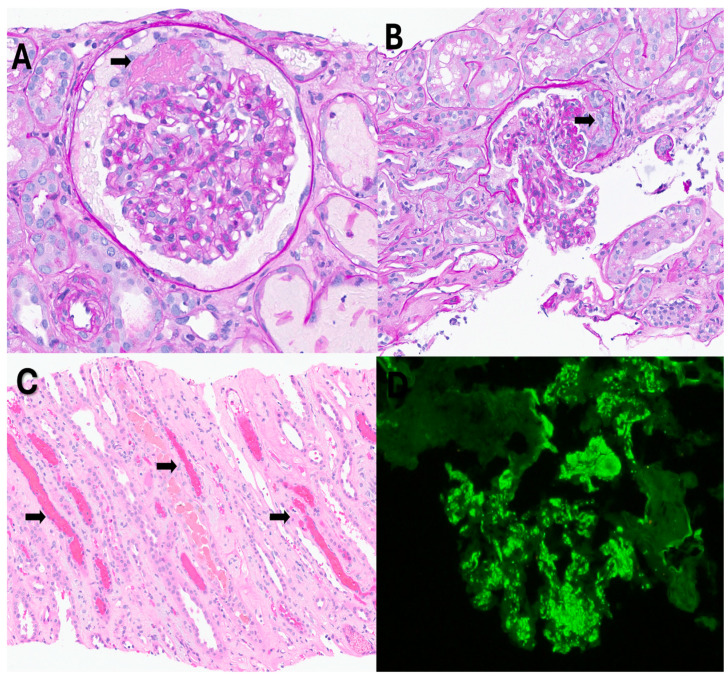
Light microscopy findings: (**A**) Glomerulus with segmental fibrinoid necrosis (arrow). (**B**) Glomerulus with segmental cellular crescent (arrow). (**C**) Red blood cell casts (arrows) in distal tubules. (**D**) Immunofluorescence histology for C3 shows granular mesangial and capillary loop staining.

**Figure 2:**
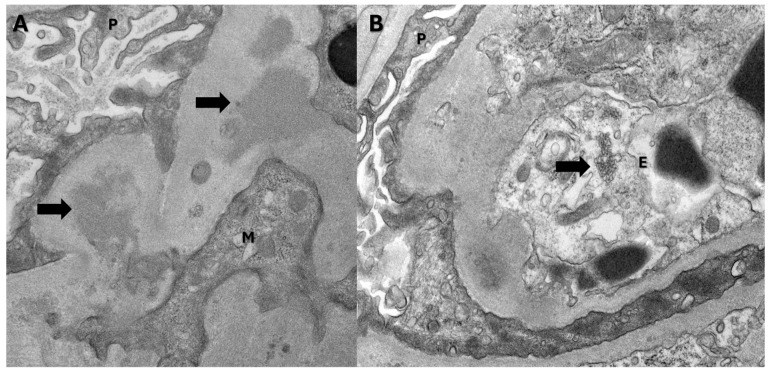
Electron microscopy findings: (**A**) Electron-dense deposits (arrows) in the mesangial region (P: visceral epithelial cell; M: mesangial cell). (**B**) Tubuloreticular inclusion body (arrow) (P: visceral epithelial cell; E: endothelial cell).

**Figure 3:**
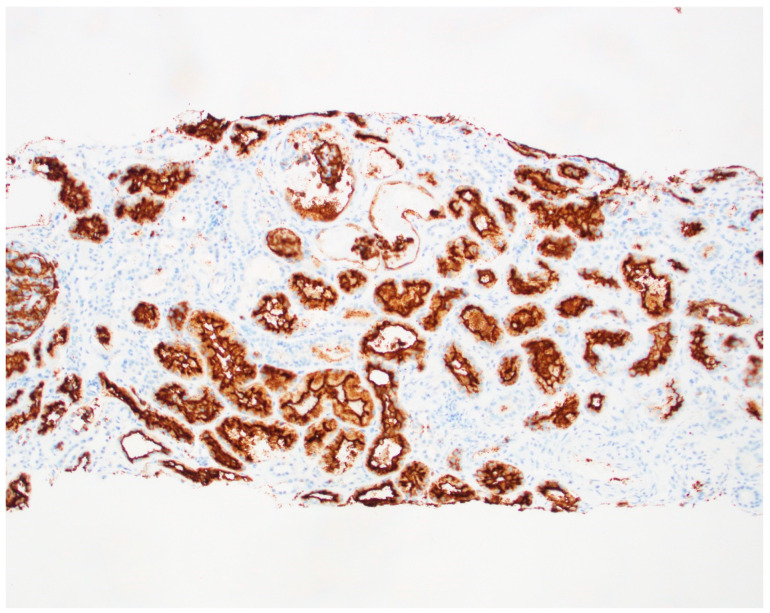
Biopsy slide with CD 10 staining of the proximal tubule.

He underwent extensive investigation. His blood and urine cultures were negative. Serologies for antinuclear antibody, anti-PLA2R (Phospholipase A2 receptor) ab, viral hepatitis panel, ANCA (antineutrophil cytoplasmic antibody) panel, cryoglobulins, complements C3, C4, anti-GBM (glomerular basement membrane) antibody, serum protein electrophoresis and serum free light chains all came back negative. He tested negative for influenza and positive for SARS-CoV-2 virus (Table S2). A SARS-CoV-2 test was performed on this patient along with other respiratory viruses—Influenza A, Influenza B and respiratory syncytial virus. These were a nasal swab collections and PCR was performed on these samples. He did not have any respiratory symptoms but had several non-specific symptoms and his chest X-ray was clear. Renal vein Doppler studies, bilateral lower extremity Doppler studies and a V/Q scan to look for any pulmonary emboli in the context of active COVID-19 infection all came back negative. The patient then underwent kidney biopsy. The biopsy showed (Figure 1 and Figure 2) focal necrotizing and crescentic glomerulonephritis with mesangial proliferative changes and C3 deposits (active COVID-19 infection-associated). We also stained the biopsy with CD10 to identify the proximal tubules (Figure 3). Kidney tissue was not stained for the SARS-CoV-2 virus as it was not available in our lab. Other causes of glomerulonephritis, bacterial infection-related glomerulonephritis and C3GN (C3 glomerulonephritis) were also considered. But his blood cultures, urine cultures and anti-streptolysin O titers came back negative, and the biopsy did not show any significant staining for IgG (bacterial infection-related glomerulonephritis usually shows strong staining for C3 and IgG). C3GN was strongly considered. His serum C3 and C4 were within normal range. We tested for complement pathway abnormalities, which came back negative. But since his biopsy showed tubular injury (usually seen with COVID-19 infection), tubuloreticular inclusion bodies (indicating high interferon activity seen with viral infections), several C3 deposits (seen due to complement activation in COVID-19 infection) and active COVID-19 infection, the patient’s condition was diagnosed as active COVID-19 infection-induced glomerulonephritis rather than C3GN. The pathologist determined that the pattern of C3 deposition and overall features of the biopsy (light microscopy, EM and IF) are not suggestive of typical C3GN but are secondary to COVID-19 infection. The patient was then started on Remdesivir (5 days), pulse steroids and an oral prednisone taper.

His renal function, hematuria and urine output gradually improved. He was in the hospital for 10 days. He was discharged with prolonged prednisone taper, with a starting dose of 60 mg daily. Four weeks later, his creatinine improved to 5 mg/dL. Eight weeks later, his creatinine improved to 2.5 mg/dL, and 3 months later, this improved to 1.8 mg/dL and stabilized at around 1.8 mg/dL; his eGFR also improved to 35 mL/min (Figures S2 and S3). He tapered down his prednisone to 5 mg over the next three-months. His follow-up urinalysis showed only 3–10 RBC per high-power field, but no WBC, blood or protein, and clear clarity (Table S3). His urine albumin to creatinine ratio improved from 249 mg/g to 17 mg/g (Figure S1). His blood pressure eventually improved, and he was maintained on atenolol 50 mg daily, hydrochlorothiazide 25 mg daily and amlodipine 10 mg daily.

## 3. Comment

The biopsy demonstrates focal necrotizing (1 of 16) and crescentic glomerulonephritis (1 of 16) associated with C3 deposits in the mesangium and capillary walls. The differential diagnosis includes infection-related versus C3 glomerulonephritis. Given the presence of ongoing COVID-19 infection, this is favored to be secondary to the infection.

## 4. Final Diagnosis

Focal necrotizing and crescentic glomerulonephritis with mesangial proliferative changes and C3 deposits (active COVID-19 infection-associated).

## 5. Discussion

The COVID-19 virus primarily infects the lungs, but several other organ systems are also affected due to it. A study published by Pei et al. from China revealed that the incidence of acute kidney injury reached up to 75% in COVID-19 infected patients [3]. The most common risk factors for COVID-19-related acute renal failure are chronic kidney disease, type 2 diabetes mellitus, hypertension, male sex, old age and the severity of COVID-19 infection [2]. COVID-19 affects the tubular, vascular and glomerular compartments of the kidneys [4]. This susceptibility is partly due to the presence of Angiotensin-converting enzyme 2 (ACE2) receptors in the kidneys, particularly in the podocyte and proximal tubule epithelial cells [2]. The spike protein on the virus interacts with these ACE2 receptors, allowing entry into host cells. Due to the high concentration of ACE2 receptors in the lungs and kidneys, these organs become primary targets for the virus [2]. The interaction between the virus and ACE2 receptors on endothelial cells and podocytes in the glomerulus can lead to hematuria, proteinuria and a reduction in the glomerular filtration rate [5]. These organs are suspected to act as the reservoir of the virus, and the detection of viral mRNA in urine specimens reported in some studies adds further support to these clinical findings [2].

Acute kidney injury associated with COVID-19 infection is a multifactorial etiology, with cardiovascular effects due to unstable hemodynamics, the direct viral impact on the kidneys, immune system dysregulation, cytokine storm, hyper coagulopathy, endothelial injury, collapsing glomerulopathy, and thrombotic microangiopathy all playing a role [6].The most common cause of AKI in COVID-19 infection is acute tubular necrosis from septic shock [2]. The virus infects proximal tubular cells, leading to vacuolar degeneration and a loss of the brush border of tubular epithelial cells, leading to the development of acute tubular necrosis [6]. Endothelial cell infection by the virus leads to the formation of fibrin thrombi in the glomerular capillaries [6]. This endothelial injury, along with a prothrombotic state, leads to small vessel vasculitis and extensive micro-thrombosis, which in turn cause thrombotic microangiopathy (TMA) and renal injury. Interleukin-6 (IL-6) is crucial in the cytokine storm syndrome observed in COVID-19 infections. The cytokine storm contributes to ARDS and impairs hemodynamics, which leads to reduced renal perfusion, renal medullary hypoxia and tubular cell damage, leading to acute tubular necrosis [6].

## 6. Various Glomerular Pathologies Seen After COVID-19

### 6.1. Infection Across Published Studies

#### 6.1.1. Podocytopathies

- Collapsing glomerulopathy (CG);
- Membranous nephropathy (MN);
- Secondary focal segmental glomerulosclerosis (FSGS);
- Global glomerulosclerosis;
- Minimal change disease (MCD);
- Primary FSGS.

#### 6.1.2. Immune Complex-Mediated Glomerulonephritis (GN)

- IgA nephropathy;
- Lupus nephritis;
- Immune complex-mediated GN with polytypic IgG3 and IgM-dominant immune deposits.

#### 6.1.3. Pauci-Immune GN

- ANCA-associated GN;
- Anti-glomerular basement membrane (anti-GBM) GN.

#### 6.1.4. Vascular Dominant

- Thrombotic microangiopathy (TMA).

#### 6.1.5. Patients with Multiple Pathologies on Biopsy

- ATN + collapsing glomerulopathy (CG);
- ATN + minimal change disease (MCD);
- ATN + membranous nephropathy (MN);
- ATN + IgA nephropathy;
- ATN + thrombotic microangiopathy;
- ATN + CG + MN;
- ATN + CG + IgA nephropathy;
- ATN + Henoch–Schönlein purpura nephritis.

Glomerular diseases associated with COVID-19 are described under an entity called COVID-19-associated nephropathy (COVAN) [7]. During the COVID-19 pandemic, several glomerular diseases were reported in association with COVID-19 infection. This is due to immune dysregulation, autoantibody production, cytokine storm, complement activation and direct viral toxicity associated with COVID-19 infection, which led to various forms of glomerular injuries [7]. The treatment of the glomerular diseases in the setting of active or recent COVID-19 infection is challenging as these diseases require immunosuppression [7].

In a review study conducted by Kudose et al. on kidney biopsy findings of patients with COVID-19-related kidney injury, patients with collapsing glomerulopathy and minimal change disease were found to have high-risk APOL1 genotypes in genetic studies, and electron microscopy showed endothelial tubuloreticular inclusions in 60% samples [8]. The presence of tubuloreticular inclusions indicate the role of interferon-mediated injury in genetically susceptible individuals with COVID-19 infection [8,9]. These inclusions, referred to as “interferon footprints,” indicate a significant role for interferon in the kidney pathology associated with COVID-19 [7]. Despite extensive investigation using multiple distinctive methods, this study did not detect any viral particles within the kidney cells [8]. Even in patients with positive COVID-19 RT-PCR (reverse transcription polymerase chain reaction), the immunohistochemical staining of the kidney biopsy samples and electron microscopy did not show any viral particles (SARS-CoV-2—severe acute respiratory syndrome coronavirus 2) in the kidney tissues [4].

PLA2R, expressed in the respiratory tract and the kidney, may trigger PLA2R-mediated membranous nephropathy if the respiratory tract is infected by SARS-CoV-2. There are case reports describing the association of membranous nephropathy and COVID-19 infection, with elevated PLA2R titters observed in these cases. Biopsies from these patients have shown features of secondary membranous nephropathy and they have responded to immunosuppression [10].

Gobor et al. described a case of crescentic membranoproliferative glomerulonephritis in a patient with a previous history of chronic membranoproliferative glomerulonephritis who had received a COVID-19 vaccine. This patient responded well to aggressive immunosuppression, which led to the normalization of renal function and an improvement in proteinuria. This report illustrates the possibility of COVID-19 vaccination triggering glomerular disease [11].

In a study conducted in South Korea by Kim et al., the most common glomerular disease diagnosed after infection with COVID-19 was podocytopathy with primary focal segmental glomerulosclerosis and minimal change disease, whereas post-vaccination, the most common glomerular diseases were IgA nephropathy and Henoch–Schönlein purpura nephritis, with a few cases of lupus nephritis and pauci-immune crescentic glomerulonephritis. COVID-19 infection and vaccination can lead to autoimmune glomerulonephritis through the activation of innate and adaptive immune responses. COVID-19 vaccinations, particularly mRNA vaccines, are believed to enhance immune reactions, which can trigger the development of glomerulonephritis [12].

Winkler et al. reported a case of recurrent anti-glomerular basement membrane disease in a patient following SARS-CoV-2 infection. This patient had negative serologies, but their biopsy showed fibrocellular crescents, segmental fibrinoid necrosis and linear IgG, IgM and C3 deposits along the glomerular basement membrane. This patient was treated with plasma exchange, pulse steroids and Rituximab, with a positive outcome. This case illustrates the possibility that COVID-19 infection can reactivate pre-existing auto reactive T lymphocytes and B lymphocytes, and can activate the complement system, leading to inflammation which in turn leads to glomerular endothelial injury resulting in crescentic glomerulonephritis [13].

Ta et al. described a case of ANCA-associated vasculitis with mucosal involvement in a patient recovering from COVID-19 pneumonia. This paper suggests that COVID-19 infection triggered autoimmunity through molecular mimicry, viral persistence, epitope spreading and the formation of neutrophil extracellular traps. These neutrophil extracellular traps are formed by activated neutrophils which lead to endothelial injury, complement activation and the production of Perinuclear-ANCA and Cytoplasmic-ANCA, which led to crescentic glomerulonephritis. They also suggest that the expression of neutrophil extracellular traps expression is increased in COVID-19 patients [14].

Pfister et al. studied kidney biopsies of patients with COVID-19 infection and discovered marked complement activation in the vascular beds and tubules. All three pathways of complement activation were observed in COVID-19 infection. Complement activation in kidneys was observed to be indirect rather than direct, as viruses were not detected in kidney tissue by in situ hybridization and immunohistochemistry in the study. Complement C3c and specifically C3d deposition was noted extensively in the tubules of patients with COVID-19 infection and the intensity of staining correlated with the severity of COVID-19 infection. C5b-9 deposits were also detected and showed high intensity staining in COVID-19 infection patients. These findings highlight the role of complement-related kidney injury in COVID-19 [15].

C3 glomerulopathy is caused by the dysregulation of an alternate complement pathway, mainly related to complement gene abnormalities. It is characterized by persistently low serum C3 and autoantibodies directed against various complement factors that can be detected in serum. It can present clinically with asymptomatic hematuria, proteinuria or severe glomerulonephritis, and can progress to ESRD (end-stage renal disease). A biopsy showed 3+ staining for C3 either in the form of sausage-shaped deposits and the thickening of the basement membrane in dense deposit disease or as electron-dense deposits in mesangial and subendothelial regions in C3GN. This biopsy picture is described as MPGN (membranoproliferative glomerulonephritis) usually. Treatment response is usually poor, but it may respond to anti-complement agents (anti-C5a agent—Eculizumab) and MMF (Mycophenolate Mofetil) [16]. In comparison, COVID-19-related glomerulopathy is secondary to COVID-19 infection. Clinical presentations are usually variable. Excessive proteinuria is usually seen in cases of collapsing glomerulopathy, which itself is seen in cases of high-risk APOL1 carriers. Biopsy findings are not consistent, unlike in C3 glomerulopathy. Acute tubular necrosis is the predominant finding in biopsies. Tubuloreticular inclusion bodies are noted in biopsies of COVID-19-associated kidney injury, indicating interferon activity which is not seen in C3GN. Complement activation is secondary to infection and serum C3 levels eventually normalize unlike in C3 glomerulopathy. Biopsies usually show C3 deposits in tubules and vascular beds and all three pathways of the complement system activated. Treatment is directed against COVID-19 with antivirals.

Yilmaz et al. reported a case of an adolescent with biopsy-proven IgA nephropathy and Alport syndrome who developed crescentic glomerulonephritis due to flare up of IgA nephropathy following COVID-19 infection. Despite immunosuppression with pulse steroids, it progressed to end-stage renal disease and the patient became dialysis-dependent. This paper shows that COVID-19 infection stimulates IL-6 production, leading to an excess production of galactose-deficient IgA1 and flaring up IgA nephropathy. Various cytokines released in response to COVID-19 infection stimulate the maturation and proliferation of IgA1-producing B cells. COVID-19 vaccination like influenza vaccination stimulates the production of IgA1 monomers, leading to IgA nephropathy flare up [17].

Duran et al. described cases of pauci-immune glomerulonephritis (ANCA-associated vasculitis) following COVID-19 infection which were successfully treated with pulse steroids, cyclophosphamide and plasma exchange [18].

Klomjit et al. describe managing collapsing glomerulopathy, membranous nephropathy, crescentic IgA nephropathy, sero-positive crescentic pauci-immune glomerulonephritis, anti-GBM glomerulonephritis and proliferative glomerulonephritis with monoclonal immunoglobulin deposits which presented in patients following COVID-19 infection and were managed with various immunosuppressive regimens which included steroids, cyclophosphamide, Rituximab, Cyclosporine and Tacrolimus along with antivirals and Plex in a few cases, with varying outcomes [7].

Bell et al. describes modifying an immunosuppressive regimen in transplant patients to improve the outcomes. These include discontinuing or holding Mycophenolate and switching to a mechanistic target of a rapamycin (mTOR)-based regimen. Patients on a CNI (calcineurin inhibitors), mTOR and prednisone regimen have shown better outcomes according to this paper [19]. Using various antivirals and monoclonal antibodies directed against COVID-19 virus early in the course of infection was also advocated in this paper [19]. Kronbichler et al. proposed that in patients with COVID-19 infection with lymphopenia and kidney transplant recipients with severe COVID-19 infection, further suppressing the T-cell immunity with immunosuppressants should be avoided. Controlling the cytokine storm and inflammatory state with anti-IL-6 agents should be considered. Kidney transplant recipients on triple immunosuppression should receive antiproliferative drugs to allow the immunity to fight the infection. Severe COVID-19 infections associated with severe lymphopenia could slow the progression of immune-mediated glomerular disease, even if immunosuppression is discontinued or not initiated [20].

The management of COVID-19-related acute kidney injury (AKI) involves treating septic shock, adapting lung-protective ventilation strategies, controlling cytokine storms and using antiviral agents targeting SARS-CoV-2. Renal replacement therapy, in cases of hypervolemia, causes refractory hypoxemia, acid-base disturbances and electrolyte abnormalities. Continuous renal replacement therapy (CRRT) helps to remove inflammatory molecules from the body and mitigate the cytokine storm [2]. Mortality rates in patients with COVID-19-related AKI correlate with viral load and AKI severity. Patients with pre-existing chronic kidney disease (CKD) required renal replacement therapy more frequently and those with advanced CKD who developed AKI often continued maintenance dialysis after discharge [2].

Ueda et al. published a case series of five patients with biopsy-proven IgA nephropathy who developed gross hematuria within 48 h of COVID-19 symptom onset, which lasted for one week. Acute kidney injury accompanied hematuria in some cases, and notably, some patients had no prior history of gross hematuria despite having IgA nephropathy [21].

In our case, the patient had no previous history of chronic kidney disease or IgA nephropathy. His hematuria was attributed to infection-mediated crescentic glomerulonephritis, secondary to COVID-19 infection. The biopsy revealed fibrinoid necrosis, crescentic glomerulonephritis, several RBC casts (in distal tubules), tubuloreticular inclusions in endothelial cells and C3 deposits supporting the diagnosis of infection-related glomerulonephritis (Figure 1 and Figure 2—biopsy images) from active COVID-19 infections. We performed the CD10 staining to identify the proximal tubules. Despite the absence of respiratory symptoms, the patient was treated with Remdesivir (an antiviral agent inhibiting SARS-CoV-2 RNA-dependent RNA polymerase), pulse steroids and a prolonged prednisone taper over three months. The treatment led to the resolution of hematuria and improved renal function. This case is unique as it represents infection-related crescentic glomerulonephritis secondary to COVID-19 presenting initially with gross hematuria, a manifestation not previously described in the literature.

## Data Availability

All data pertaining to this study are presented in the manuscript.

## Supplementary Materials

The following supporting information can be downloaded at: https://www.mdpi.com/article/10.3390/jcm14103302/s1, Figure S1: Albumin/creatinine trend over time; Figure S2: creatinine trend over time; Figure S3: eGFR variation over time; Table S1: Complete Blood Count (CBC) Results Summary; Table S2: Infectious and Autoimmune Serology Results Summary; Table S3: Urinalysis Results Summary.
