# Supplementary material for: Crescentic Glomerulonephritis Possibly Caused by COVID-19 Infection
Source: J Clin Med. 2025 May 9;14(10):3302. doi: 10.3390/jcm14103302 (PMC12112333; doi:10.3390/jcm14103302)
Supplement: Supplementary file 1 [file jcm-14-03302-s001.zip › jcm-3578591-supplementary.pdf]

Journal of Clinical Medicine

MDPI

Supplementary Table S1. Complete Blood Count (CBC) Results Summary.

Test Name	ED initial presentation	Follow up Out-Patient	Hospital
Hemoglobin (g/dL)	16.0	11.4 (L)	11.9 (L)
Hematocrit (%)	44.6	31.3 (L)	34.7 (L)
Erythrocytes (million cells/μL)	4.94	3.61 (L)	3.64 (L)
MCV (FL)	90.3	86.7	95.3
RBC Distribution Width	11.4 (L)	11.0 (L)	12.9
Platelet Count (cells/μL)	171	189	226
Leukocytes (cells/μL)	6.9	8.4	7.2
Neutrophils (cells/μL)	3.94	7.51 (H)	-
Lymphocytes (cells/μL)	2.22	0.74 (L)	-
Monocytes (cells/μL)	0.64	0.18 (L)	-
Eosinophils (cells/μL)	0.04	<0.03	-
Basophils (cells/μL)	<0.03	<0.03	-

Supplementary Table S2. Infectious and Autoimmune Serology Results Summary.

Test Name	Result
Influenza A, PCR	Undetected
Influenza B, PCR	Undetected
Respiratory Syncytial Virus, PCR	Undetected
SARS Coronavirus-2, PCR	Detected
Antinuclear Ab Screen by IFA, S	Negative
Anti PLA2 R Ab	< 2 RU/ml
Glomerular Basement Membrane IgG Ab	<0.2
Myeloperoxidase Ab, S	<0.2
Proteinase 3 Ab (PR3), S	<0.2
Complement C3, S (mg/dL)	113
Complement C4, S (mg/dL)	24
Kappa Free Light Chain, S	8.01 (H)
Lambda Free Light Chain, S (mg/L)	3.88 (H)
Kappa/Lambda FLC Ratio (mg/L)	2.06 (H)
Total Protein, S (g/L)	5.7 (L)
Albumin Electrophoresis	3.3 (g/L)
Alpha-1 Globulin	0.2
Alpha-2 Globulin	0.7
Beta-Globulin	0.7
Gamma-Globulin	0.7
A/G Ratio	1.39
Impression	No apparent monoclonal protein on serum electrophoresis.

HBs Antibody, S	Negative
HBs Antigen, S	Nonreactive
HCV Ab Screen, S	Negative
Anti streptolysin-O Titer, S (Todd Units/mL) Normal value < 160	110 Todd units/ml
Alternative Complement Path Functional, S (> or = 46%)	80%

Supplementary Table S3. Urinalysis Results Summary.

**Test Name**	**ED initial presentation**	**Follow-up Out-Patient (1 week)**	**Hospital Day 1**	**Hospital Day 2**	**Post-Hospitalization (1 month)**	**Post-Hospitalization (2 month)**	**Post-Hospitalization (3 month)**
Color, U	Orange	Dark	-	Amber	yellow	-	Yellow
Specific Gravity	1.021	1.008	-	1.020	1.010	1.005	1.010
Protein Urine Random(mg/l)	>=300	>=300	-		44	88	Negative
Blood	Large	Large	-	Large	Moderate	Moderate	small
White Blood Cells (WBCs/hpf)	21-30	>100	-	>100	25-50	10-20	Occ-3
Urine RBC (RBCs/hpf)	>100	>100	-	>100	10-20	3-10	3-10
Albumin/Creatinine Ratio (mg/g)	-	249 (H)	162 (H)	118 (H)	94 (H)	68 (H)	17

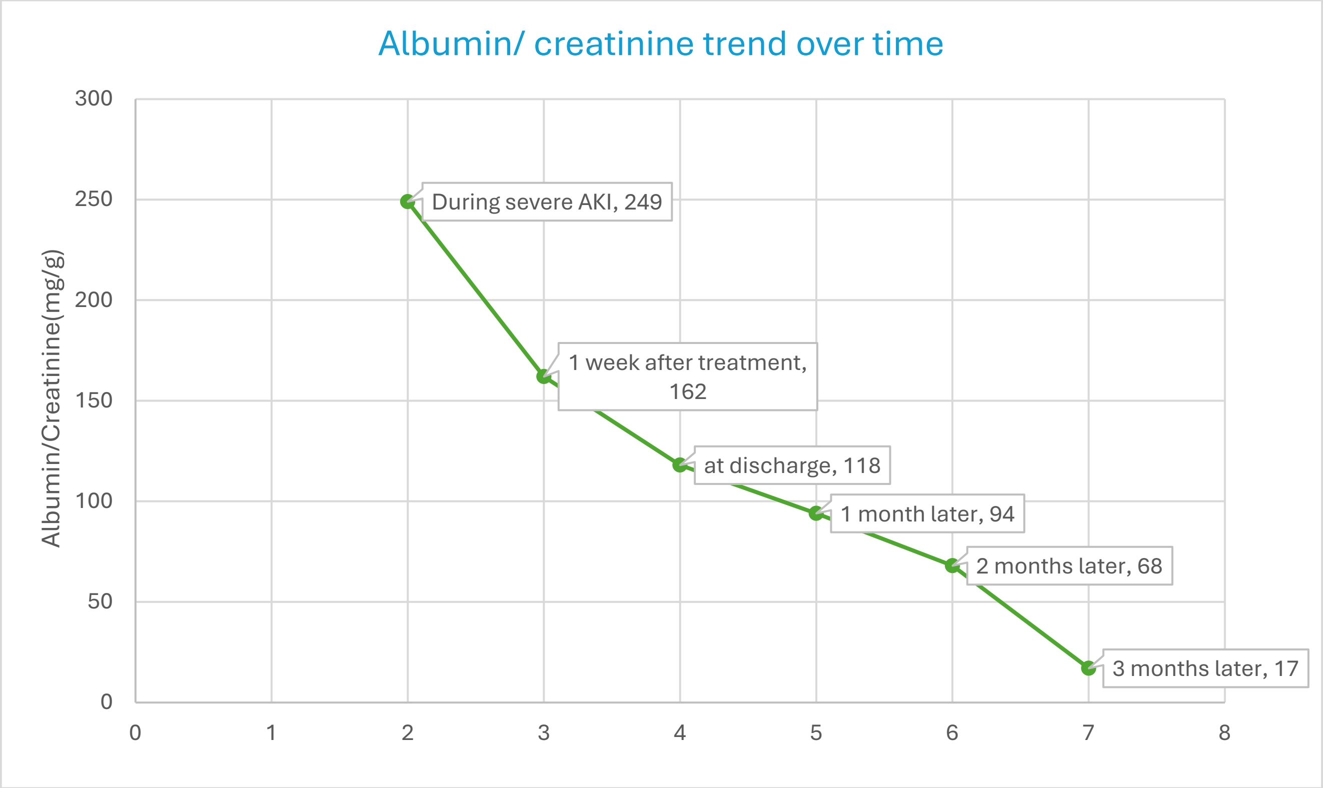

Supplementary Figure S1. Albumin/creatinine trend over time.

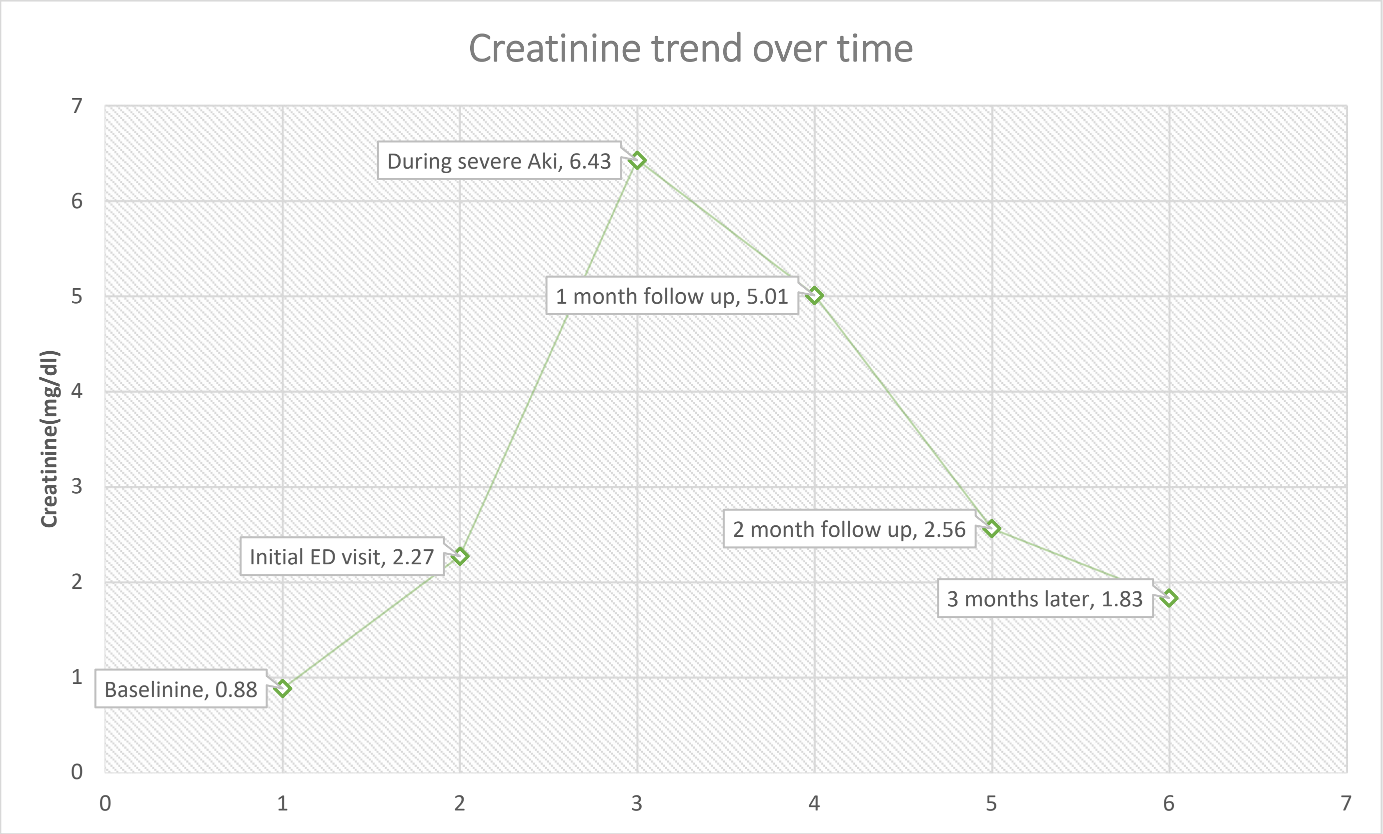

Supplementary Figure S2. creatinine trend over time.

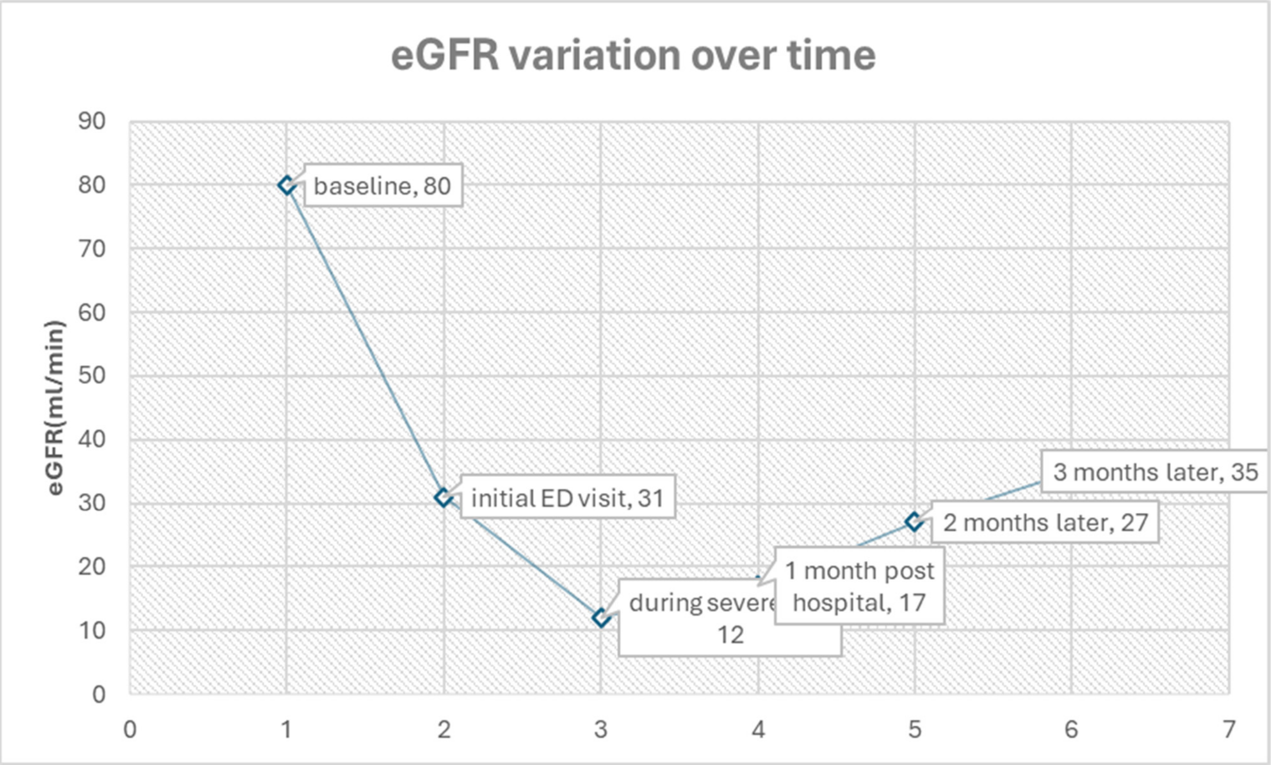

Supplementary Figure S3. eGFR variation over time.
